# Conventional and Recent Trends of Scaffolds Fabrication: A Superior Mode for Tissue Engineering

**DOI:** 10.3390/pharmaceutics14020306

**Published:** 2022-01-27

**Authors:** Islam M. Adel, Mohamed F. ElMeligy, Nermeen A. Elkasabgy

**Affiliations:** Department of Pharmaceutics and Industrial Pharmacy, Faculty of Pharmacy, Cairo University, Kasr El-Aini Street, Cairo 11562, Egypt; m.f.elmeligy@gmail.com (M.F.E.); nermeen.ahmed.elkasabgy@pharma.cu.edu.eg (N.A.E.)

**Keywords:** tissue engineering, scaffolds, biomaterials, fabrication, organ-on-a-chip

## Abstract

Tissue regeneration is an auto-healing mechanism, initiating immediately following tissue damage to restore normal tissue structure and function. This falls in line with survival instinct being the most dominant instinct for any living organism. Nevertheless, the process is slow and not feasible in all tissues, which led to the emergence of tissue engineering (TE). TE aims at replacing damaged tissues with new ones. To do so, either new tissue is being cultured in vitro and then implanted, or stimulants are implanted into the target site to enhance endogenous tissue formation. Whichever approach is used, a matrix is used to support tissue growth, known as ‘scaffold’. In this review, an overall look at scaffolds fabrication is discussed, starting with design considerations and different biomaterials used. Following, highlights of conventional and advanced fabrication techniques are attentively presented. The future of scaffolds in TE is ever promising, with the likes of nanotechnology being investigated for scaffold integration. The constant evolvement of organoids and biofluidics with the eventual inclusion of organ-on-a-chip in TE has shown a promising prospect of what the technology might lead to. Perhaps the closest technology to market is 4D scaffolds following the successful implementation of 4D printing in other fields.

## 1. Introduction

The survival of living organisms depends mainly on their self-healing capabilities in response to tissue damage. Tissue damage refers to any alteration in the structure of a tissue, being a hard or soft one. Hard tissues include bones and teeth, while soft tissues refer to any tissues connecting and supporting different body structures and organs, such as ligaments, muscles, and tendons. Tissue damage could be brought by chemical, mechanical, or even pathological causes. To reverse tissue damage, our bodies are programmed to initiate a self-healing mechanism known as ‘tissue regeneration’. For example, in skin injuries, such an auto-repair mechanism takes place in three successive and overlapping steps, namely, hemostasis and inflammation, new tissue proliferation, and finally, new tissue maturation and remodeling [1,2]. During the inflammatory stage, blood clotting cascade takes place immediately to stop the bleeding. Chemical mediators and cytokines are released to increase vascular permeability and attract neutrophils to remove dead cells and foreign bodies. Fibroblasts are attracted to the injury site through the release of platelet-derived growth factor and fibrin, then collagen synthesis begins [3]. In the proliferative stage, re-epithelization along with vascularization take place, along with continued proliferation of the fibrin network [4]. Finally, in the remodeling stage, the formed matrix gains strength, and excess collagen is degraded [5]. However, when the damage is so severe that the body’s own self-healing mechanism cannot cope with the rate of cellular demise, or when the tissue is of the non-replicating type, tissue/organ transplantation is the sole solution. However, several limitations arise with transplants, including the limited numbers of donors and possible transplant rejection [6]. Such limitations had paved the way to look for a more feasible approach serving as a complementary system to conventional therapy. One proposal involved the regeneration of new tissues in place of the defective ones to restore normal tissue/organ function. This marked the introduction of the concept of ‘tissue engineering (TE)’. TE dates to 1933, when mouse tumor cells were first incorporated within a polymeric membrane into the abdominal cavity of a pig, where they avoided the detection by the immune system and remained viable [7]. The successful incorporation of tumor cells led to the introduction of artificial organs later in 1964 [4]. However, the modern concept of TE was later introduced by Langer and Vacanti in 1993 as “an interdisciplinary field that applies the principles of engineering and life sciences towards the development of biological substitutes that restore, maintain or improve tissue functions” [8]. Moreover, they shed light on isolated cell implants, incorporation of growth factors to promote tissue regeneration, and use of matrices to carry cells, growth factors, and signaling cues to the defect tissue [8]. The incorporation of cells, drugs, and/or biological factors into supporting matrices (scaffolds) represented a scientific breakthrough in the modern TE. There are mainly two approaches for TE using scaffolds: ex vivo ‘top-down’ and in situ ‘bottom-up’ approaches [6,9].

It is worth mentioning that TE can be based on scaffold-free technology. For example, Haraguchi et al. developed a temperature-responsive culture surface known as ‘cell sheet technology’, in which cells are attached in layer-by-layer form and fused together [10]. They used a culture surface modified with a temperature-responsive material such as poly(N-isoproplyacrylamide) (PIPAAm), where attachment and detachment of cells can be controlled by altering temperature. The technology proved beneficial in many cases such as esophageal ulceration to replace missing epithelial tissue [11], cardiac tissue to replace damaged myoblasts [12], and pancreatic islet cells in diabetes mellitus [13]. However, the technology is still far from optimized, particularly for complex tissues, and the interference of interdisciplinary fields is a must to advance [14].

A different method to obtain scaffold-free constructs is to pile up spheroids (aggregates of cells) in a mold and culture them until maturation to fuse together and form a construct taking the shape and dimensions of the mold [15]. The method is simple and free of the complex environment conditioning in ex vivo scaffold-based TE and can be used in non-replicative tissue, unlike the in situ approach; however, this approach is accompanied by a risk of immunogenicity and infection as well as safety of degradation byproducts in the long term [15].

In this review, attention will be directed to the more mainstream scaffold-based approaches with design considerations, biomaterials used, and different conventional and advanced fabrication techniques employed to produce scaffolds.

## 2. Approaches for Scaffold-Based Tissue Engineering

### 2.1. Ex Vivo Tissue Engineering

In the ex vivo approach, stem cells are isolated from the donor and seeded on/within a scaffold. Following this, the cell-laden scaffold is exposed in a suitable environment in bioreactors to specific signaling cues that promote the proliferation and formation of the desired tissue type. The cultivated scaffold is later implanted into the target tissue. Consequently, the implanted scaffold must be fabricated to be of the exact shape and size of the affected tissue it replaces. At last, scaffold degradation takes place to allow substitution with the newly formed tissue and avoid any incompatibility with the recipient immune system [16]. Although this approach allows the use of a wide array of biomaterials and provides scaffolds of optimum mechanical properties [16], many hurdles limit its application. For instance, the selective differentiation of cells into the desired tissue is an extremely difficult task to begin with since it requires the careful mimicking of the niche internal environment together with the autocrine and paracrine signaling mechanisms involved in the process [17]. There is also the problem of limited availability of acceptable donor tissue biopsies either due to the immune-compatibility requirement between the donor and recipient immune system or due to tissue morbidity under artificial conditions in the bioreactors [9]. Moreover, the prefabricated scaffolds lack any intrinsic bioactivity. This was solved by the preceding addition of bioactive materials, hence, further complicating the conditioning of the niche microenvironment [18,19].

### 2.2. In Situ Tissue Engineering

Drawbacks of the ex vivo approach forged the way to seek an alternative technique that is far less complicated. In situ TE emerged as a more convenient solution to promote tissue regeneration. In this approach, scaffolds of the required shape, size, and properties are prefabricated with suitable biophysical and/or biochemical cues, implanted directly into the affected area without the obligation of prior seeding with cells. Following so, both the immuno-compatibility concerns and the complicated conditioning for prior cell culturing were both avoided. The implanted scaffolds attract the host’s own endogenous cells to the injury site and promote tissue regeneration [20]. Obviously, the in situ TE approach cannot be used for the regeneration of non-regenerative tissues since it relies on the body’s intrinsic regenerative ability [9]. In some instances, the resulted tissue suffers from poor mechanical properties due to the lack of control over cellular differentiation and assembly, unlike in the ex vivo model [21].

**Figure 1:**
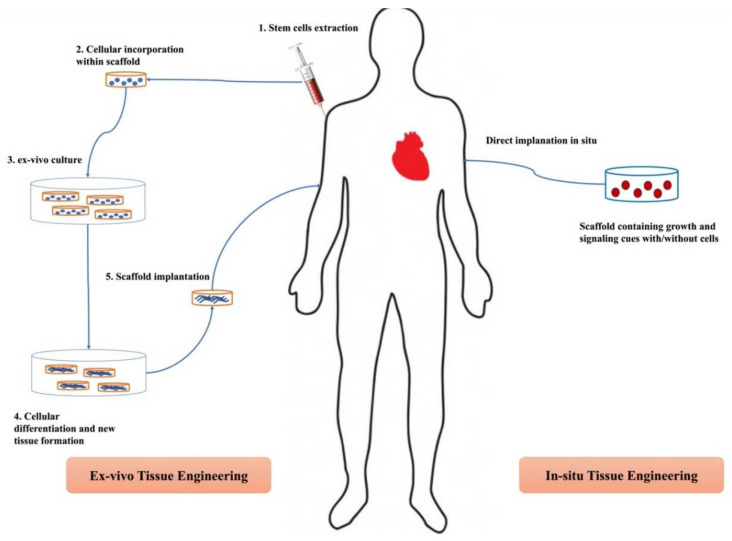
A schematic illustration of ex vivo and in situ TE approaches in scaffold-based tissue engineering focusing on the basic steps being followed.

Figure 1 illustrates the differences between the ex vivo and in situ TE approaches.

## 3. Scaffolds and Tissue Engineering

Initially, scaffolds were solely used as supporting matrices. However, as the field of TE advanced over time, other functions had emerged. For instance, by carrying appropriate growth factors and signaling cues, they could signal cell differentiation and facilitate tissue regeneration [22]. Incorporating drug molecules within the scaffold could be one way to directly deliver the drug to the targeted injury site in suitable amounts [2].

Scaffolds possess various biological, structural, and chemical properties that need to be carefully tuned according to the properties of the affected tissue. This is easily achievable via selecting the appropriate fabrication technique. For example, the electrospinning technique is best suited for cases for implantation in tissues where high flexibility is required, such as in soft tissues [23]. Different fabrication methods will be addressed later in this review, but first, we will shed light on principal scaffold features to be taken into consideration when designing scaffolds as well as biomaterials used in the fabrication process.

### 3.1. Features of Scaffolds

**Figure 2:**
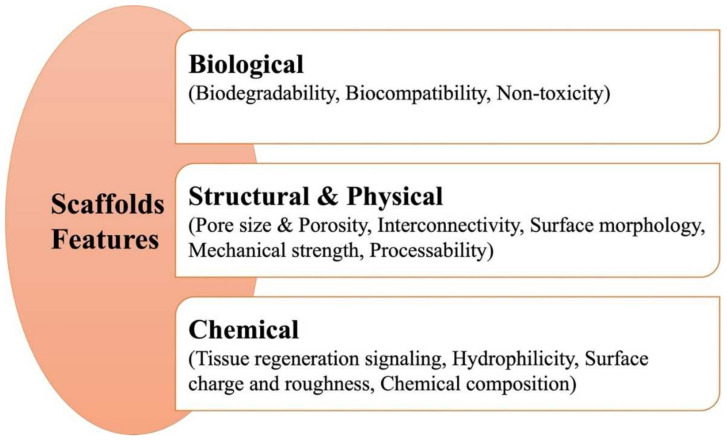
Features to be considered for optimal scaffold design and fabrication. Customizing the scaffold features is conceptualized according to the target tissue and the required aim.

When designing scaffolds, several design decisions should be made with respect to the required scaffold features, depending on the target site and the needed function. These features can be categorized into biological, structural, and physical considerations as well as chemical aspects. A summary of principal features can be seen in Figure 2.

#### 3.1.1. Biological Concerns

Biological concerns are related to how an implanted scaffold would affect the biological system. Scaffolds should be biocompatible, biodegradable, nontoxic, and mimic the properties of the extracellular matrix (ECM) of the original tissue [6,24]. Biocompatibility is of extreme importance to cell growth and successful tissue regeneration, as any sort of incompatibility will interfere with cellular ability to regenerate new tissues [24]. Scaffolds should degrade either spontaneously or by the action of enzymes normally present at the target site [25] while leaving nontoxic byproducts in the process. The degradation process may occur at a rate either equal to the rate of new tissue formation or slower to ensure proper tissue healing [9,26,27]. For example, Mann et al. demonstrated that hydrogels based on the photopolymerizable polyethylene glycol (PEG) derivatives with proteolytically degradable peptides in their structure underwent lysis by collagenase and elastase enzymes [28].

#### 3.1.2. Structural and Physical Considerations

A living tissue is a complex three-dimensional (3D) structure. Since scaffolds should perfectly mimic the target tissue to ensure efficient tissue healing, certain structural and physical considerations are required in scaffold design; otherwise, alterations in the niche tissue environment would occur [9]. Porosity, mechanical behavior, pore size, and pore interconnectivity (channel connecting pores), as well as surface topography, are examples of structural and physical consideration.

Scaffold porosity refers to the percentage volume occupied by the voids within a scaffold in respect to the bulk volume of the scaffold [29]. High porosity is a favorable attribute for tissue regeneration. High porosity enhances oxygen diffusion, water transport, and nutrient supply as well as facilitates cellular infiltration thus, aiding in tissue regeneration [2,30,31,32]. However, very high porosity values are not recommended, as this implicates poor mechanical properties [33]. Davidenko et al. stressed that a careful balance should be attained between porosity and mechanical strength during their studies on collagen/hyaluronic acid 3D scaffolds [34]. A proper balance between mechanical strength and porosity plays a vital role in chondrogenesis as well as ligaments and muscles formation. This has to do with the influence they exert over spatial organization and differentiation as well as the degree of stretching of the formed tissue [35]. Sussman et al. also demonstrated the effect that the optimum pore size has on the spatial organization of various macrophage responses for scaffolds implanted subcutaneously in mice [36]. The authors carried their research on non-porous, 34 μm-porous implants and 160 μm-porous implants. In 34 μm implants, reduced fibrosis and enhanced angiogenesis were observed, typical of increased M1 cells (M1; classically activated macrophages responsible for pro-inflammatory cytokines release [37]) residing within the pores of the implants. In 160 μm implants, the presence of M2 cells (M2; alternatively activated macrophages responsible for the up-regulation of CD200R membrane glycoprotein [37]) resulted in improved healing. They concluded their work highlighting the effect of pore size where larger pore sizes provide spacious room for macrophages to self-adhere without interacting with the pore wall. Interaction with pore walls would cause the downregulation of M2 cells. Consequently, the pore size should be optimized to ensure the initiation of the inflammatory stage (characteristic of M1 cells) and the healing stage (characteristic of M2 cells).

In different works, the authors varied the pore topology via using different block copolymers as emulsion stabilizers. They found that surface topology, together with porosity and interconnectivity, strongly affected mesenchymal cells attachment, a prerequisite for cellular differentiation and tissue regeneration [38].

Another noteworthy concern is the processability of scaffolds. Scaffolds need to be fabricated into different shapes and sizes feasibly, with the fabrication process being cost-effective [39].

#### 3.1.3. Chemical Aspects

When designing scaffolds, careful attention should be given to the events following the implantation step. Implanted scaffolds should not be only fully capable of predestining the surrounding microenvironment for new tissue formation but also ensuring that the signaling cues will be released properly. After all, signaling cues are the main stone for TE. They include growth factors, proteins, drugs, etc. Their main role is to signal many functions, including cellular infiltration and differentiation, angiogenesis, receptor-mediated responses, and even initiating scaffold dissolution and degradation [9].

On the other hand, chemical modification of scaffold surface with different functional groups can modify cellular adhesion through altering surface hydrophilicity, surface charge, chemical composition, and/or surface roughness [40].

Increasing surface hydrophilicity is a popular approach for modifying a scaffold’s surface. One way to achieve that is via oxygen-plasma (O_2_ plasma) treatment. One study highlighted the effect of O_2_ plasma treatment on increasing fibronectin (a glycoprotein that binds to ECM proteins) adsorption to scaffolds, which eventually caused a subsequent increase in osteoblasts attachment [41]. The direct relation between the enhanced cellular attachment to surfaces with pre-adsorbed fibronectin was well-illustrated elsewhere [42]. The contact angle was used as a measure of wettability and surface hydrophilicity, where a decrease in contact angle indicated an increase in both terms.

Alteration of surface free energy is of key importance in titanium-based dental implants. Many untreated titanium oxide (TiO_2_) surfaces are characterized by low surface energy either due to their hydrophobicity or their tendency to adsorb hydrocarbons from the surrounding environment [43]. On the other hand, hydroxylated TiO_2_ led to the phenotypic expression of different osteoblasts exhibiting higher surface energy and increased alkaline phosphatase and osteocalcin activities [44]. The former is a bone mineralization regulator, while the latter is an osteoblast differentiation marker [44].

According to chemical composition, scaffolds could be prepared using polymers, bioceramics, metallic biomaterials, or hybrid/composites of two or more biomaterials [31,45].

### 3.2. Biomaterials in Scaffolds Fabrication

Biomaterials choice is extremely crucial in TE. Biomaterials should comply with general properties such as biodegradability, biocompatibility, and non-toxicity, as well as site-specific ones. For example, for soft tissues such as cartilage or muscles, an easily processible material is clearly chosen over a stiff one. Below are the main classes of biomaterials used in TE, highlighting major characteristics and differences.

#### 3.2.1. Metallic Biomaterials

Investigation of metals in TE dates older than other biomaterials [46]. Owing to their excellent physical and mechanical properties, metals were investigated in dental and bone TE [47]. Metals can be biodegradable or non-biodegradable. Among the non-biodegradable metals used are titanium (Ti), tantalum (Ta), stainless steel, and alloys such as titanium-nickel (Ti-Ni) and cobalt-chromium (Co-Cr) alloys, while biodegradable metals include iron (Fe), magnesium (Mg), and zinc (Zn) alloys [48].

In fabricating metal-based scaffolds, many challenges are faced. The biodegradation kinetics of the metals should be heavily assessed, particularly for resorbable metals. The rate of degradation should match the rate of new tissue formation; otherwise, loss of mechanical strength could occur prior to restoration of tissue function [49]. Another major consideration is the elastic modulus of the used metal. The difference in elastic moduli between the bone and the metal should be kept to a minimum; otherwise, stress shielding would cause implant loosening and eventual implant failure [50]. Surgical intervention to implant placement and removal, which is not only invasive but also can expose the patient to the risk of infection, remains the main limiting concern to the use of metal implants [51]. Overall, metals should be nontoxic, non-allergenic, biocompatible, and with suitable wear and corrosion resistance. Metal wear and tear cause the leaching of metal ions, which, when they exceed their permissible levels, cause inflammation and tissue lesions [52].

To alter the properties of metals or impart new function(s), surface modification can be carried out. Ti is known to have excellent tensile strength while being biocompatible, which rationalizes its use in load-bearing implants [49]. However, being bioinert with poor osseointegration hinders its cell interaction capability and biomedical application [50]. Through the incorporation of titania nanotubes on the Ti surface, the nanotube-modified Ti promoted bone marrow mesenchymal cells adhesion, osteogenic differentiation as well as the antibacterial activity of Ti [51]. Additionally, it was observed that the success rates of the prepared matrices relied mainly on the diameter size of the incorporated nanotubes.

Despite their limitations, metals remain valuable, particularly in orthopedic applications. However, precise tailoring of the metal used, according to the desired profile and acceptable limitations, is needed when selecting the appropriate candidate.

#### 3.2.2. Natural Polymers

Natural polymers are those obtained from natural renewable sources such as animals, plants, algae, and other microorganisms [52,53]. They are favorable options in TE for a number of reasons. They perfectly mimic the ECM impose certain biological activities with acceptable porosity, all while possessing excellent biodegradability and biocompatibility profiles [16,35,54,55]. Natural polymers often have intrinsic bioactivity, thus aiding in accelerating tissue regeneration [56]. However, many disadvantages have directed researchers into developing synthetic biomaterials. Being of natural origins, they may contain unwanted impurities, show inter-batch variations, have difficult processability due to complex structures, and may illicit immunogenic reactions [35,41,57].

They could be classified according to their structures into polypeptides (fibrin, collagen, gelatin, and keratin), polysaccharides (starch, cellulose, hyaluronic acid, chitosan), and polynucleotides-based polymers (DNA and RNA) [58]. Depending on the required property, polymer choice differs. For instance, fibrin shows minimal immunogenic reactions and is completely biodegradable; however, its poor mechanical strength limits its application in hard tissues [59]. Alternatively, silk fibroin (SF) exhibits excellent mechanical properties, but having a slow rate of degradation presents a major concern in scaffold fabrication [60]. In an attempt to overcome this, Kim et al. prepared hydrogels using methacrylated SF, which possessed a vastly enhanced degradation rate as compared to non-modified SF [61]. Switching to hyaluronic acid, its in vivo metabolites have angiogenic characteristics, which could come in handy in TE, particularly when biodegradability, biocompatibility, and non-immunogenicity are considered [62]. However, its high viscosity and high-water retention render its processability troublesome [63]. In a previous work conducted by our research team on fabricating HA-based curcumin wafers, the authors found that the high-water retention capability of HA caused rapid degradation of wafers when compared to non-HA-based wafers [2]. This was attributed to the extreme hydrophilicity and the hydrogen bond interaction with water molecules in the surrounding media, causing the rapid dissolving of the wafers.

#### 3.2.3. Synthetic Polymers

Perhaps the lack of predictability and poor processability of nature polymers held them back as biomaterials for TE. In fabricating scaffolds that mimic the complex 3D structure of the living tissues, synthetic polymers became the go-to option. This is mainly due to the flexibility by which they are processed, the variety of shapes they can be manipulated into, the ease by which their properties can be modified as well as the lack of immunogenic reactions [64,65,66,67]. A key obstacle in using synthetic polymers is their poor biological activities and cellular affinity. Both are attributed to the lack of functional groups, which renders their modification a difficult task [68]. Advancements in science introduced new synthetic polymers known as ‘functional polymers’ to overcome the limitations of conventional synthetic polymers. Functional polymers have unsaturated bonds and/or functional groups in their structure that tailor them for different needs [67,69,70]. Aliphatic polyesters such as polyglycolic acid (PGA), polylactide-co-glycolide (PLGA), polylactide (PLA), and poly(*ε*-caprolactone) (PCL) represent the most used synthetic polymers in TE [35,63]. Other synthetic polymers include polyhydroxybutyrate (PHB), poly (ester amide) (PEA), polyethylene glycol (PEG), polyurethanes (PU), and polyvinyl alcohol (PVA) [57,63,71].

As mentioned earlier, synthetic polymers can be easily modified to alter their properties as needed. PHB, despite being difficult to process with its poor mechanical profile, is one of the most used polymers in scaffolds intended for bone TE [72,73]. By adding 3% *w*/*w* alumina to the PHB-chitosan alloy solution, the produced scaffolds gained a 10-fold increase in the tensile strength compared to the plain alloy [73]. In another study, the poor mechanical profile of PHB-gelatin nanofibers was significantly improved following the inclusion of collagen in the mix [74]. PCL has several advantages in TE, including biocompatibility, ease of processability, and stability under normal conditions. However, due to its hydrophobicity and poor wettability, it exhibits poor cellular adhesion behavior [75]. Air plasma treatment of PCL and PCL-hydroxyapatite (HAp) nanofibers improved their wettability and hydrophilicity, as indicated by the massive reduction in water contact angle. This, in turn, resulted in enhanced cell adhesion and proliferation [76].

It can be concluded that advances in synthetic polymers and the introduction of functional polymers had them tailored for the construction of different scaffolds with vast physicochemical properties by tuning their structure, further strengthening the potential of synthetic polymers in TE.

#### 3.2.4. Bioceramics and Bioglass

Bioceramics include a large group of inorganic biomaterials with suitable biocompatibility, excellent mechanical profiles, and high melting points, which render them suitable in orthopedic and dental TE [77,78,79]. However, they are brittle and thus, cannot be used in load-bearing situations. To overcome this, they are usually combined with certain polymers [78]. Ceramics can be categorized based on their tissue response into; bioinert ceramics such as alumina and zirconia, bioactive ceramics such as glass ceramics, and finally, biodegradable ceramics such as calcium phosphates. HAp and beta-tricalcium phosphate (β-TCP) are the most used calcium phosphates owing to their excellent osteoconductivity and biocompatibility [79,80,81]. While bioinert ceramics cannot create stable bonds with the tissue, both bioactive and biodegradable ceramics allow bond formation, with biodegradable ones having the added benefit of degrading over time as they are being replaced by the newly formed bone tissue [82]. One parameter is of great value when choosing calcium phosphates is the calcium: phosphate ratio (Ca: P). Ceramics with Ca: P < 1 are not biologically favored, while those with Ca: P > 1.67 tend to resorb slowly [83].

The merging of nanotechnology with the use of Zn-HAp ceramics for the drug delivery of doxorubicin was proven successful in targeting post-operative cancer tissues. The ceramic implant (Zn-HAp) ensured the targeting part while the incorporated drug-loaded nanoparticles enabled the enhanced drug release as well as boosted drug uptake kinetics. Overall, Zn-HAp ceramic showed excellent results against MG-63 osteosarcoma cell lines [84].

One way to improve the osteogenic abilities of bioceramics is to include silica ions in their construction. Silica ions improve osteogenesis through activation of multiple gene-transduction pathways, which imparts osteoinductive effect to the bioceramic [85]. The combination of silica ions with calcium and phosphate to improve silica reabsorption is known as ‘bioactive glass’ [86]. Bioactive glasses can bind strongly to bone tissue and, upon exposure to physiological conditions, they form a superficial HAp layer on the target site and induce bone tissue regeneration [87,88]. Bioactive glass can be manufactured using either the melt quenching technique or the sol-gel transition method [82]. Many bioactive glasses are available with large variations in their mechanical strength. For example, 45S5 Bioglass^®^ is brittle while CEL2 and SCNA were optimized to reach much higher compressive strength values at 5–6 MPa and 15 MPs, respectively [89,90].

Attempts to enhance the mechanical strength and biological properties of bioglass were made by doping several metal ions individually or in combination into the bioglass. Metals used include strontium, iron, manganese, etc. These dopants can be selected according to the body minimum requirements of such elements. This can assist in the repair process [91].

#### 3.2.5. Clay Minerals

Clary minerals refer to a class of materials belonging to the phyllosilicates and composed mainly of sheets of tetrahedral silicates and octahedral hydroxides blocks, ranging in size from nano to micro range and exhibiting permanent surface charge [92,93]. Those silicate sheets are fixed together via many sorts of interactions, and, accordingly, clay minerals can be classified into several classes [92,94]. In the case where the clay is composed of one tetrahedral sheet and one octahedral sheet, we refer to classes such as kaolinites and serpentine, while if the sheets are one octahedral and two tetrahedral, we have groups such as smectites, chlorite, vermiculite, bentonite, and hectorite [93].

The combination of clay minerals and nanotechnology is visualized in layered double hydroxides nanoparticles (LDHs). LDHs are two-dimensional (2D) hydrotalcites with particle sizes up to 100 nm with an overall positive charge under acidic pH [95,96]. LDHs have many advantages that attracted much research over the past few years. These include biocompatibility, biodegradability, low toxicity, high drug loading capacity, controlling drug release, anionic exchange properties, and antibacterial activity [97]. In a study performed by Li et al., the antibacterial activity of penicillin G was prolonged when prepared as penicillin G-loaded Zn-Al LDHs [98].

Another example is halloysite nanotubes (HNTs) fabricated from the halloysite of the kaolinite group. Porous nanocomposite scaffolds composed of agarose, gelatin, chitosan, and HNTs were prepared via freeze-drying and doped with allogenic mesenchymal stem cells (MSCs) [99]. When tested for the repair of a dog bone defect, the added HNTs were found to impart osteoinductive effect to the prepared scaffolds. Table 1 highlights some of the biomaterials used in TE with key advantages and disadvantages.

### 3.3. Approaches to Scaffolds Optimization

**Figure 3:**
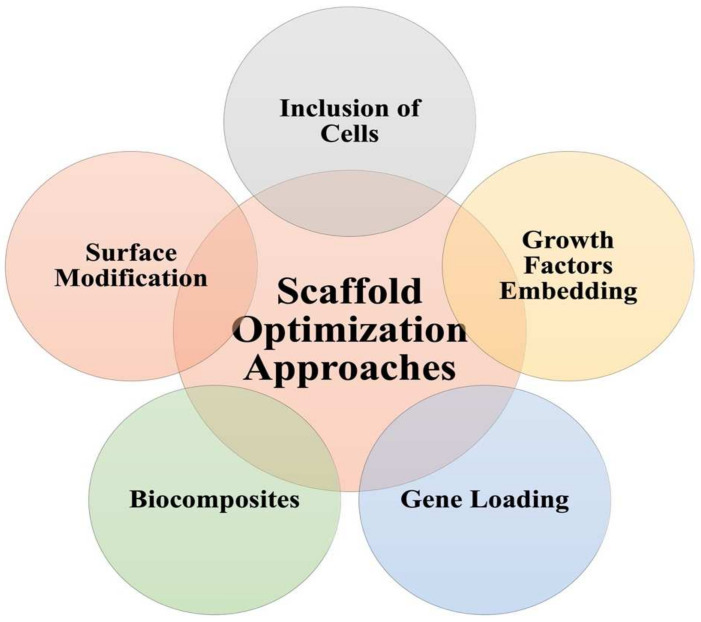
Different approaches used to optimize scaffolds functions. Introducing functional groups on scaffolds surfaces can enhance active therapeutic moieties loading as well as the interaction with targeted tissues. Therapeutic moieties include drugs, growth factors, and genes that can promote the scaffold functionality in addition to augmenting cellular proliferation. Inclusion of cells into the scaffolds can achieve the formerly mentioned benefits. Tailoring of scaffolds design and characteristics is also achieved through the formation of biocomposites prepared by blending different types of biomaterials from various origins.

Biomaterials represent the backbone of scaffolds structure; however, they are not solely responsible for the function of scaffolds. To overcome the challenges facing the successful implementation of scaffolds, several approaches are proposed, as shown in Figure 3.

Surface modification is one way of scaffolds optimization. It can be carried out via techniques such as chemical or plasma treatment, aiming at introducing new functional groups. Such new groups could aid in the interaction with ECM or allow new drugs incorporation [120]. During their studies on bone tissue regeneration, Gentile et al. were able to improve bone mineralization using surface-modified peptides (heparin-bound) in comparison to unmodified peptides [121]. Briefly, the authors carried out plasma treatment to introduce carboxyl functional groups on the surface of nanocomposite made of a new polymer combination between polyhedral oligomeric silsesquioxane (POSS) and polycarbonate-based urea–urethane (PCU) (POSS-PCU nanocomposite). Following, the modified nanocomposite was coupled using carbodiimide chemical reaction with heparin-bound peptides to induce osteoblast attachment and differentiation. ALP assay was carried out on both the protein-bound nanocomposite and the unmodified nanocomposite, where a 2.7-fold increase in ALP activity of bone marrow mesenchymal stromal cells was noticed in the modified nanocomposite.

The inclusion of cells within the scaffold matrix is another optimization technique to enhance scaffold function and promote tissue proliferation. Osteoblasts, the main bone-forming cells, and chondrocytes, the main cells in hyaline cartilage, can be incorporated in scaffolds suited for bone and cartilage regeneration, respectively. After their extraction and in vitro culture, they were found to be preprogrammed to carry out their tasks and initiate bone and cartilage tissues differentiation [122]. Lack of cellular sources of osteoblasts and chondrocytes was an issue until solved via using MSCs (adult stem cells capable of differentiating into different types of cells) [123]. MSCs can be harvested from bone marrow, the synovium, adipose tissue, dental pulp, etc. [122]. Others have investigated the use of pluripotent stem cells (undifferentiated embryonic stem cells); however, there is an unethical limitation of using human embryos [124]. To overcome this, Takahashi and Yamanaka were able to obtain induced pluripotent stem cells (iPSCs) first from adult mice and later from humans by genetic transfection using retroviruses [122,125].

Growth and signaling factors are another class of additives that enhance cellular differentiation and tissue regeneration. In one study, the authors successfully prepared HA-based poly-d,l-lactic acid/polyethylene glycol/poly-d,l-lactic acid (PDLLA-PEG) hydrogel loaded with both transforming growth factor-beta 3 (TGF-β3) and MSCs [126]. TGF-β3, together with MSCs, enhanced chondrogenesis while the inclusion of HA resulted in a controlled release of TGF-β3. This allowed a uniform glycosaminoglycan deposition while retaining the mechanical strength of the constructs.

Gene loading could help enhance the functional status of scaffolds. Genes are to be first introduced into the nuclei of cells, using viral or non-viral vectors, to be translated later into functional proteins at the target site, which will accelerate tissue repair [122]. Gene loading represents a favorable alternative to the short-lived growth factors [127]. A novel injectable nanoscale calcium sulfate/alginate paste was loaded with bone morphogenetic protein 2 (BMP2)-gene-modified MSCs and evaluated against MSCs-only non-BMP2-gene-loaded paste in terms of osteogenic activity [128]. The results showed robust osteogenesis, and consistent bone defect healing in favor of the BMP2-gene loaded paste.

Another approach in refining and optimizing the properties of scaffolds can be through blending different biomaterials to obtain new material of the required properties. Such blends are known as ‘Biocomposites’ [129,130]. Biocomposites can be broadly classified based on the origin of the biomaterial into natural-natural and natural-synthetic composites. Chitosan was blended with gelatin successfully and investigated in TE, where chitosan was found to promote the cell adhesion and biological activities of gelatin. On addition of silk fibroin to the blend, mechanical strength was increased while the degradation rate of the scaffold was reduced [131]. On another note, methyl cellulose was added to the chitosan/polyvinyl alcohol blend to impart plasticity to the implant to enable its use in skin TE [132].

## 4. Fabrication Techniques

**Figure 4:**
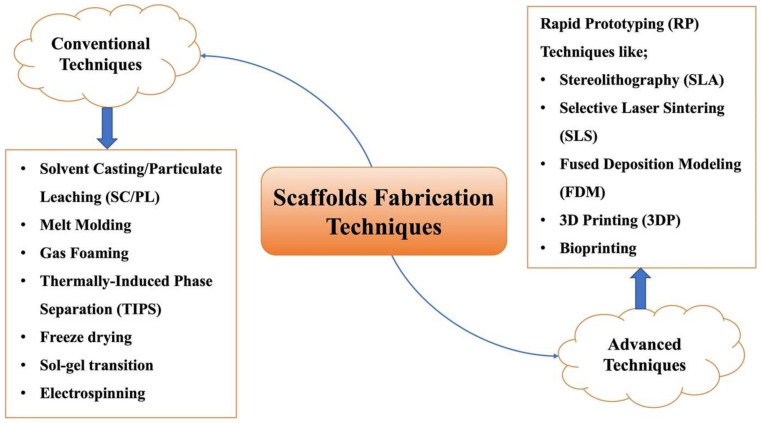
Classification of the numerous techniques that can be used in scaffolds fabrication into conventional and advanced techniques challenges and benefits of any of the mentioned techniques should be addressed prior to the scaffold fabrication to maximize patients’ benefits.

Due to the large variation in biomaterials, the intended use of scaffolds as well as the targeted tissues; hence, various fabrication techniques can be used. Fabrication techniques can be broadly classified into conventional and advanced techniques. Each technique has its merits and drawbacks. As mentioned earlier, pore size, interconnectivity as well as pore volume are essential scaffold properties that determine cell culture and new tissue fate. Pore and interconnect sizes are vital for cellular penetration into scaffold matrices as well as for the secretion of ECM. An improvement in ECM secretion, as well as uniform cell distribution, can be seen in increasing pore and interconnect sizes [133,134,135]. Depending on the target tissue, the scaffolds properties should match tissue requirements, and consequently, an appropriate fabrication method is recommended. Different fabrication techniques can be seen in Figure 4.

### 4.1. Conventional Fabrication Techniques

#### 4.1.1. Solvent Casting and Particulate Leaching (SC/PL)

In this technique, the polymer is dissolved in a volatile solvent where a porogen is uniformly distributed. Then, the solvent is evaporated, resulting in the formation of a polymer network in which the porogen is entrapped. Finally, the porogen is leached by immersing the scaffolds in a suitable porogen solvent, leaving pores within the matrix [136,137]. Usually, the used porogens are either organic compounds such as gelatin and collagen or water-soluble inorganic salts such as NaCl [138].

SC/PL is a simple, reproducible procedure that produces scaffolds with a high degree of porosity [139] as well as allows for fine adjustment of pore size through varying salt/polymer ratio, particle size, or shape of the used porogen [140,141]. However, the residual solvent and/or porogen content should be taken into consideration [142]. The limited mechanical properties, the lack of control over interconnectivity, the long processing time, and the production of thin films are other drawbacks of the method [79,143,144].

A modified SC/PL was attempted using PCL as the sole polymer as well as NaCl and PEG as dual porogen to be used in bone TE [145]. The resulted scaffolds were highly porous with suitable water absorption capacities typical of SC/PL. The added PEG, however, resulted in uniform pore size and high interconnectivity. The optimized scaffolds produced significant bone ingrowth when tested in vitro in mice, calvaria-derived, pre-osteoblastic cells (MC3T3-E1).

#### 4.1.2. Melt Molding

Melt molding is a straightforward conventional technique based on the use of thermoplastic polymers. The polymer is first melted then casted in a mold of suitable 3D structure according to the defect tissue [146]. Porosity could be introduced via merging melt molding with other methods such as particulate leaching, gas foaming, phase separation, etc., and finally, the scaffolds are usually freeze-dried [147].

The method is simple, allows individual control over morphological characteristics such as pore size and interconnectivity, and avoids the use of organic solvents. However, the elevated temperatures used in the melting might not be suitable for every component such as thermolabile drugs. Moreover, there is the problem of residual porogen if used to induce porosity [141,147].

An example of the method can be found in the work carried out by Oh et al. [148]. PLGA/PVA-based porous scaffolds, cultured with chondrocytes, were fabricated using melt molding then implanted in skull defects of rabbits to be evaluated for their bone ingrowth capabilities. The PVA-treated scaffolds showed better bone ingrowth activity as compared to the non-PVA-treated scaffolds, as implied by MTT assay and SEM findings. The improvement in bony tissue growth was attributed to the increased hydrophilicity of the PVA-treated scaffolds and hence, better cytocompatibility.

#### 4.1.3. Gas Foaming

In the conventional gas foaming technique, two approaches can be followed. The difference between both approaches depends on the method of introducing the gas. The blowing gas is either generated in situ (chemical blowing agent) or directly blown (physical blowing agent) in a polymer or a polymer-surfactant solution. On gas evolution, it leaves voids in its place, forming a porous polymeric matrix. Gases as nitrogen (N_2_) and carbon dioxide (CO_2_) are used in the first approach, while pressurized gases such as methane/hydrogen mix (CH_4_/H_2_) are used in the second one. Surfactants are present to aid in the formation and stabilization of the resultant foam [135,149].

It is apparent that gas foaming avoids the use of organic solvents; hence, hazards related to residual solvents are eliminated. Other advantages of the technique include obtaining highly porous scaffolds, minimal loss of encapsulated bioactive species, mild production conditions, and suitability for both hydrophobic and hydrophilic polymers [135,150]. However, poor interconnectivity, lack of precise control over pore size, and long operating times are serious limitations to the method [57,151,152]. It is worth mentioning that efforts are made to offer precise control over pore sizes and interconnectivity. Examples include manipulating temperature and pressure within the apparatus vessel, polymer type/concentration, using gas/organic solvent mixtures as well as applying microfluidics in an advanced gas foaming technique [135,149,153].

The anticancer gemcitabine was impregnated in foam scaffolds fabricated using PLGA as a polymer and supercritical CO_2_ as the pressurizing gas where the scaffolds possessed very high impregnation efficiency (>90%) [154].

#### 4.1.4. Thermally Induced Phase Separation (TIPS)

The core principle of TIPS is the conversion of a stable polymer system into a thermodynamically unstable one so that it is separated into two phases: a polymer-rich phase and a polymer-deficit one [155]. Briefly, a pre-heated polymer solution is exposed to low temperatures. Low temperature acts as a trigger that converts the system into the thermodynamically unstable state, inducing phase separation. Later, the solvent is removed by sublimation, extraction, or any other technique where the polymer-rich phase forms the matrix and the polymer-poor one forms the pores within the matrix [138,156]. Drugs incorporated are added to the polymer solution or with other miscible solvents prior to the solidification step.

Since it relies on low temperatures and complete removal of solvent after solidification, TIPS is suitable for bioactive drugs, particularly heat-sensitive ones [141]. It is worth mentioning that TIPS could be combined with other techniques such as SC/PL, electrospinning, 3D printing, etc. [157,158]. The process is inexpensive, allows the fabrication of highly porous scaffolds (>95%) with high interconnectivity, as well as the feasibility by which morphological properties of the produced scaffolds can be tuned [158]. This is possible through manipulation of the process parameters such as polymer type and concentration, solvent/non-solvent ratio, cooling rate, and presence of surfactant [159]. A major setback of TIPS is the use of organic solvents that needs to be completely removed to avoid their hazardous outcomes [159].

In a study conducted by Si et al., they prepared chitosan/collagen scaffolds using TIPS to be assessed in peripheral nerve regeneration [160]. The prepared composites showed superior mechanical properties and reduced degradation rate when compared to the collagen-only scaffolds, while porosity and water uptake, despite being lower, remained within acceptable values. When the composite scaffolds were tested against Schwann cells, they showed enhanced cellular adhesion and proliferation while lacking any cytotoxicity. Their in vivo findings confirmed modulation of neural degradation behavior with the absence of inflammatory reaction suggesting their potential in nerve regeneration.

#### 4.1.5. Freeze-Drying

Freeze-drying can be used to produce scaffolds or to dry preformed constructs. The method involves four successive steps [161,162]. Firstly, a polymer solution/dispersion, along with drugs and other additives, is first prepared. The used polymer could be dissolved in either organic solvent alone (non-emulsion-based freeze-drying) [141] or in organic solvent-water emulsion (emulsion-based freeze-drying) [163]. Furthermore, water-insoluble polymer could be instead suspended in water [164]. Depending on the nature of the drug, it can be dissolved/suspended in either phase. Secondly, the solution/dispersion is mold-casted then frozen below its triple point (the temperature at which the three states of matter co-exist) by liquid nitrogen, refrigeration, etc. In the third stage, primary drying takes place to remove most of the formed ice crystals via sublimation. Finally, the remaining ice crystals are removed during the secondary drying phase. On sublimation, pores are formed in place of the previously formed crystals [165].

Freeze-drying has several advantages. It is a common method that produces completely or nearly dry, highly porous scaffolds with high interconnectivity [165]. Pore sizes in the formed scaffolds could be controlled by altering the process parameters such as temperature, rate of drying, and polymer concentration [166,167]. Restrictions to large-scale scaffolding using freeze-drying are related to the high cost of operation and lengthy preparation times [168].

Several studies succeeded in preparing freeze-dried scaffolds. Composite scaffolds, made up of keratin, fibrin, and gelatin, were fabricated using freeze-drying and used for the drug delivery of the antibiotic mupirocin [169]. The fabricated scaffolds were evaluated as wound healing dressing. SEM micrographs confirmed high porosity of the resultant scaffolds (77%) while maintaining suitable mechanical properties, as evident by tensile strength testing. Cell line studies on NIH 3T3 fibroblasts and human keratinocytes (HaCaT) showed enhanced cellular adhesion and proliferation as compared to the control (untreated cells). Similarly, testing of antimicrobial activities against *S. aureus* and *E. coli* showed prominent zones of inhibition in the case of the medicated scaffolds.

#### 4.1.6. Sol-Gel Method

This method is particularly useful in the fabrication of bioceramics and bioactive glasses. It consists of many steps, as follows. Inorganic or organic metal compounds are dispersed in water where hydrolysis and polycondensation take place, turning the system into a colloidal state. The formed system can be easily casted into the defined molds where 3D network formation and gelation start through various interactions between the components. The casted dispersions are allowed to dry in the mold. Gentle heating is applied to solidify the preformed matrix. Chemical stabilization or dehydration could be carried out to produce ultra-stable bioceramics [151,170].

Attractions of the technique are summed in the low temperatures used and the chemical homogeneity of the ceramics. Moreover, it is an effective method for delayed drug delivery. However, the high cost of the raw materials and the long processing times are the main limitations to the process [79,171].

β-TCP scaffolds were prepared using the sol-gel technique, where Pluronic F127 (a non-ionic surfactant) was used as a template [172]. The microporosity increased with a fall in the sintering temperature, while a decline in Pluronic F127 concentration caused the increase in macropores sizes as well as the evolution of nanoscale grooves. Overall, immersion studies in simulated biological fluids showed the bioactivity of the scaffolds suggesting their potential use in bone TE.

#### 4.1.7. Electrospinning

Electrospinning is one of the most used conventional techniques used to produce nanofibers (NFs). The device is composed of a syringe pump, a metallic needle (spinneret), a high-voltage power supply, and a collector [173]. An electric charge is imparted to the polymer solution by means of the attached power supply, as it is being pumped via the syringe pump through the spinneret’s nozzle. Due to the potential difference between the charged polymer solution and the oppositely charged collector, the solution heads from the syringe toward the collector, where it is collected in the form of NFs [174]. The exact mechanism of NFs formation can be illustrated as follows. Above a certain voltage, the accumulated charge on the surface of the polymer solution exceeds its surface tension. This causes the elongation of polymer solution as it escapes the spinneret and, due to the volatility of the used solvent, it evaporates, causing the deposition of the spindle-shaped NFs at the collector [175]. Processing parameters such as the flight time (time during which the solution passes from the spinneret to the collector, controllable via distance control), volatility of the solvent, needle tip size, voltage applied, and geometry of the collector could be manipulated to produce NFs of the desired porosities and morphological characteristics [151,176]. Combining various biomaterials to overcome their individual limitations [177], as well as incorporation of bioactive materials, are both feasible with electrospinning [178].

Electrospinning yields NFs of high surface area/volume ratio, which, when combined with the tunability of scaffold morphological properties such as pore size and mechanical strength, demonstrates the benefits of the process in TE [179]. However, electrospinning is not applicable to all polymers, and residual solvent may remain and affect the biological attributes of the scaffolds [180].

In one study, gelatin/PCL fibers were prepared using electrospinning and investigated for the delivery of various drugs such as simvastatin and vancomycin as well as for fluorescein isothiocyanate-bovine serum albumin [181]. While gelatin improved the osteoblast cellular adhesion and proliferation as visualized by increased ALP activity and bone mineralization, PCL enhanced the mechanical strength of the fibers and imposed a controlled release pattern for hydrophilic and hydrophobic drug molecules.

### 4.2. Advanced Fabrication Techniques

#### 4.2.1. Rapid Prototyping (RP)

RP (referred to as solid free-form fabrication; SFF) techniques are based on computer-aided design (CAD) programs that design and construct scaffolds in a layer-by-layer, reproducible, and completely controlled manner [137]. RP methods are, in fact, additive manufacturing (AM) approaches since raw materials, being solid powders or liquids, are added and solidified in a layer-by-layer manner.

Advantages offered by RP techniques include the shorter time needed to reach satisfactory prototypes as well as the reduced trial-and-error stage in scaffold design and construction [79]. However, the toxicity of binder liquids imposes health restrictions, and poor resolution of the techniques (50–300 μm) limits their usage in constructs requiring fine microstructure features [182].

RP techniques are either based on laser technology or assembly techniques, and they include the likes of stereolithography, selective laser sintering, and fused deposition modeling.

#### 4.2.2. Stereolithography (SLA)

SLA was the first laser-based, 3D printing-based technology to be introduced by Charles Hull in 1986 [183]. In SLA, a photosensitive liquid resin is irradiated by a UV light beam and allowed to deposit and solidify over a moveable platform forming the first layer. Once completely solidified, the platform is lowered, and the process is repeated for several layers until the desired prototype is obtained. After the process is finalized, the uncured resin is washed-off, and the prototype is further treated via UV exposure to obtain a fully cured product [184,185].

Although the process is easy and yields 3D constructs with fine details, the processing time increases dramatically as resolution increases. Plus, many of the solidified polymers are non-biodegradable and may present toxicity hazards [179,186].

Le Guéhennec et al. fabricated calcium phosphate-based pellets with or without HAp using SLA and evaluated them for their in vitro biocompatibility with MG-63 osteosarcoma cell lines and, for their in vivo osseointegration [187]. In vitro findings confirmed the non-cytotoxicity of the fabricated pellets; furthermore, the in vivo testing revealed the close contact (adhesion) between the pellets and the new bone tissue with excellent osseointegration.

#### 4.2.3. Selective Laser Sintering (SLS)

SLS is the most popular RP laser-based technology [168]. It uses powerful laser beams to fuse and bind powdered particles together in a layer-to-layer manner based on a computer software-assembled 3D model [188]. Similar to SLA, a 3D multilayer porous structure is obtained through the deposition of multilayers using a moveable platform. The structure shows a low degree of compactness among layers deposited in the sintering procedure, which induces the required porosity [168].

SLS produces scaffolds of high porosity and pore interconnectivity in shortened processing time. However, it is difficult to remove the uncured powder, and the method struggles to create small details such as sharp corners and boundaries [182,189,190].

SLS technique was used to prepare iron (III) oxide (Fe_2_O_3_)-doped DP-Bioglass for alveolar bone regeneration post dental implantation [191]. The bioglass proved non-cytotoxic following WST-1, Live/Dead, and JC-1 stains. As it slowly degraded, it caused the subsequent release of calcium, phosphate, iron, and silica ions, all of which promoted alveolar bone mineralization as seen by xylenol orange staining. Even more, the released ions enhanced osteogenesis following the induction of genes responsible for ALP, collagen type-I, and Runx2.

#### 4.2.4. Fused Deposition Modeling (FDM)

FDM is an assembly-based technology of 3D model fabrication. In FDM, a support material is first deposited onto an established base then the main building material is allowed to deposit over the building material and solidify to take the final 3D shape. The moveable base is then lowered to allow more layers to build on top of each other in a similar manner [192,193].

Heat applied to provide a semi-molten polymer along with inconsistent pore openings limit its application to thermoplastic polymers. FDM, however, can produce scaffolds with a wide range of porosities and pore sizes [179,194].

FDM can be combined with other techniques. As an example of this, FDM was used to fabricate PCL/PLA scaffolds that exhibited a strengthened mechanical profile. Both supercritical CO_2_ and breath figures mechanisms were employed to enhance the porosity of the FDM-fabricated scaffolds [195].

#### 4.2.5. Three-Dimensional Printing (3DP) and Bioprinting

3DP is another laser-based technology that uses a CAD model to obtain a 3D structure. A 3D ink-jet printer is used to distribute a layer of powder on the moveable platform. Following, droplets of a liquid binder are jetted over the preformed layer to bind the particles together. Different layers are deposited above each other in a similar manner. Finally, unbound powders are removed, and a 3D model is obtained [39,196].

3DP is fast emerging owing to its cost-effectiveness and rapid conversion rates of CAD files into 3D constructs. The technique is widely versatile and can be used in the production of constructs with fine details; however, FDM uses a binder liquid that requires its complete removal post processing, which is a tedious and often incomplete process [197,198].

Porous titanium alloy-based scaffold, Ti-6Al-4V, was fabricated using 3DP and tested in vivo for possible tendon fixation following prosthetic implantation. Micro-CT and hard tissue staining visualized the increased fibroblasts adhesion to and growth into the 3D-printed scaffolds of 527.15 μm pore size [199].

Bioprinting is an extension of 3DP used to produce pre-tissues, tissues, or complete organs based on the same technique implemented in 3DP [200]. Different approaches are used in bioprinting, including autonomous self-assembly and biomimicry for full organs and small building blocks for pre-tissues and tissues. Bioprinting can either be used to produce cellular or acellular constructs, with acellular ones being the easier approach. The omission of cells in acellular constructs means fewer restrictions during the manufacturing process [201]. Bioprinting, by fabricating 3D organs, indeed could be the solution to the limited supply of organ transplants [202,203].

Advanced techniques are not in the early stages anymore to be used in TE as sole replacements to the conventional methods. Even more, the benefits added, particularly overcoming cell supply shortcomings and the much shorter time needed to generate a full tissue of an organ, are worthy of research and development.

## 5. Types of Scaffolds

Different fabrication techniques produce scaffolds of different characteristics. This mandates that the selection of the technique is made according to the tissue site and the required properties. Saying so, there are various types of scaffolds to be highlighted.

### 5.1. Nanofibrous Scaffolds (NFs)

Elongated, fiber-like scaffolds with very large length (width at the nano range) are referred to as ‘nanofibrous scaffolds’. There are many techniques used to prepare NFs, including phase separation, self-assembly, template synthesis, melt is blown, etc. [177,204,205]. Each method has its limitations. For example, phase separation yields fibers that lack structural stability [206], template synthesis is a time-consuming process [207], and self-assembly forces narrow biomaterial choices [208]. The most suited technique used in NFs fabrication is electrospinning. Electrospinning allows the production of NFs with high porosity, high interconnectivity, and with large surface area as well as for the precise control over NFs structure and diameter [209,210,211]. Various biomaterials are suited for NFs manufacturing using the electrospinning technique. These include natural polymers such as collagen and CS, synthetic polymers such as PCL, PLA and PGLA, and biocomposites such as CS/ PCL and CS/SF [205].

### 5.2. Hydrogel-Based Scaffolds

Hydrogels, being injectable, 3D printed, or porous [45], refer to networks formed through chemical or physical crosslinking between hydrophilic polymers, either of natural or synthetic origins [212]. Generally, chemically crosslinked hydrogels are more favorable as they offer better mechanical resistance and wider control over scaffold properties than physically crosslinked ones [213]. A key advantage in hydrogel scaffolds over other types of scaffolds lies in their swelling ability when subjected to an aqueous environment, as the case within living tissues, rendering them perfect replicas of normal ECM. Perhaps the reason for this has to do with the hydrophilic nature imparted by hydrophilic groups (carboxyl, hydroxyl, amine, etc.) present within their structure that facilitates interaction with water molecules in the surrounding environment [214]. Hydrogels can act as support networks in TE, drug carriers, and release mediators, as well as being accelerators of wound healing through retention of nutrients and promotion of angiogenesis [2,215,216]. Hydrogels can be prepared via SC, gas foaming, freeze-drying, electrospinning, 3DP, etc. [214], and biomaterials used can be natural such as chitosan, gelatin, and HA or synthetic such as PLA, polyvinyl alcohol (PVA), and PEG derivatives [217].

### 5.3. Microsphere-Based Scaffolds

As the name suggests, microspheres are free-flowing particles in the micron range (1–1000 μm) capable of encapsulating bioactive molecules and releasing them in a controlled manner [218]. In microsphere-based scaffolds, microspheres serve as the building blocks of the scaffold. This is due to the feasibility by which microspheres can be packed with, either alone or with other biomaterials, to form 3D matrices [219]. They can be classified as either injectable or sintered scaffolds and are usually prepared by RP techniques (such as SLS), emulsion solvent extraction method, TIPS, subcritical CO_2_ sintering, etc. [219], with biomaterials such as chitosan, collagen, PLGA, alginates, etc., being used in the fabrication process [220,221,222,223].

## 6. Therapeutic Application of Scaffolds in Tissue Engineering

Scaffolds may be used to load active moieties and directly deliver them to the target tissue/ organ. Examples from the literature for therapeutically active scaffolds to be used in the treatment of several conditions are mentioned in Table 2.

## 7. Futuristic Directions in Tissue Engineering

**Figure 5:**
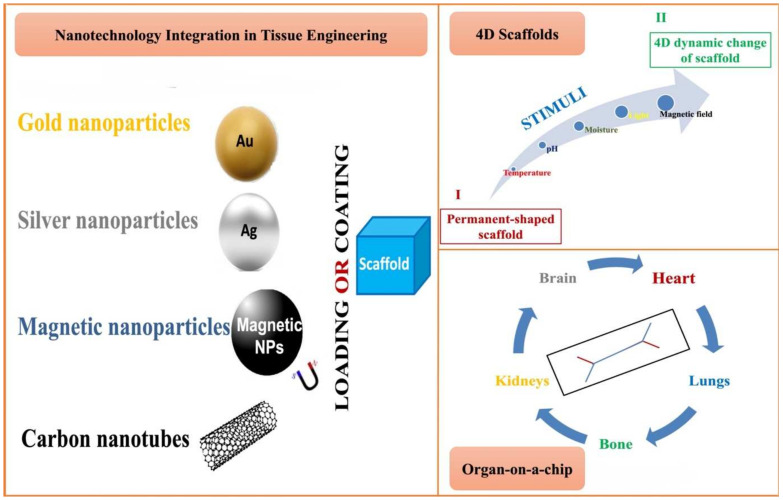
Examples of visionary approaches in scaffolds and the field of tissue engineering. Merging nanoscience and tissue engineering has a significant impact in the healthcare sector. Combining the best of both enhanced the scaffolds properties for therapeutic and diagnostic purposes. Four-dimensional printing is the next-generation fabrication strategy of patient-specific scaffolds fusing the 3D printing outcomes of producing a 3D model with definite properties with the benefits of smart materials imitating the dynamic reaction of the tissues to any external stimuli. The organ-on-a-chip approach is considered the answer to the hurdles related to simulating in vivo conditions. The clinical translation of these futuristic approaches is highly required.

The future of scaffolding and TE is promising, with advances in biomaterial science and new fabrication techniques being validated. Now, several technologies are being investigated prior to market release. In this section, we will provide brief ideas regarding some of those technologies, which are schematically presented in Figure 5.

### 7.1. Nanotechnology and Tissue Engineering

Nanoparticles (NPs) are defined as minute entities ranging in size between 1 and 1000 nm; thus, having a large surface-to-volume ratio, which facilitates cellular penetration and interaction with living cells [230]. When used in TE, NPs can serve many functions depending on their type. Among those use scenarios, NPs are used to control scaffolds properties for imaging purposes, as well as for gene, growth factors, and drug delivery [231]. Nanocoating of biomaterials can improve their biocompatibility and surface adhesion [232]. Gold NPs (GNPs) were found to enhance scaffolds’ electrical properties in myocardial TE and to promote osteogenic differentiation and osteoblast formation in bone TE [233,234,235].

PVA/GNPs composite scaffolds were fabricated using 3DP and used for the delivery of ampicillin in orthopedic postsurgical infections, where they proved to be osteoinductive, biocompatible, and with suitable antimicrobial activities [236].

Silver NPs (AgNPs) were shown to possess antimicrobial activity besides improving the mechanical properties of the fabricated scaffolds [237,238].

Magnetic NPs (MNPs) are iron oxides, particularly Fe_2_O_3_ or Fe_3_O_4_ [231]. With the aid of an external magnetic field, MNPs could target specific tissue, plus, they could be used in cell imaging and MRI applications [239,240].

Carbon nanotubes (CNTs), on the other hand, can alter the electromechanical profile of the targeted scaffold [241].

NPs present tremendous potential in TE. However, their tendency to accumulate in the body for a long period mandates extensive toxicity, carcinogenicity, and teratogenicity studies if large-scale clinical application is desired.

### 7.2. 4D Printing (4DP) and Smart Biomaterials

The output of 3DP is a 3D structure printed using data provided by CAD. Such constructs do not undergo any dynamic changes post printing. In a constantly evolving world, as technology advances, dynamic changes in the 3D-printed structure in response to an external stimulus are achieved. 4DP refers to the printing of a 3D structure assisted by CAD; however, this 3D structure undergoes dynamic changes influenced by one or more external stimuli, owing to the presence of biological moieties known as ‘Smart/responsive biomaterials’ [242,243]. In other words, the main advantage of 4DP over 3DP is that it is capable of mimicking not only the structure of the organ but also its dynamic function as it changes over time in response to stimuli such as temperature, pH, moisture, light, magnetic field, etc. [244].

The prospects of using smart biomaterials in the biomedical field are growing. An example of a stimulus-response action is visualized in exploiting the new tissue formation as a stimulus to cause gradual degradation of the implanted scaffold or biomedical device, leaving space for newly formed tissue [245]. The degradation of the scaffold/device ensures complete tissue restoration [246].

Research on 4DP is still scarce, particularly those with in vivo studies. Among those is a study carried out by Miao et al. where they managed to produce shape memory polymer-containing, PCL-based 4D scaffolds [247]. The 4D scaffolds achieved dynamic shape changes with 100% recovery under physiological conditions. In vivo findings showed enhanced human bone marrow-derived MSC adhesion and proliferation when compared to PCL control.

In another research, Wang et al. formulated a shape memory hydrogel composed of Pluronic F127 diacrylate macromer (alginate) with 3DP implied the successful implementation of the 4DP approach [248]. Alginate was responsible for the shape memory function (response) of the hydrogel when chelated with calcium ions (stimulus). The formed hydrogel was not only found to undergo shape fixation or recovery on interaction with or substitution of Ca^+2^, in that order, but also the internal structure and drug release behavior were altered. When compared to traditional 3D-printed hydrogel, the shape memory hydrogel was able to show a 1.6-fold increase in the amount of drug released over a 6 h period. Such enhanced release could be beneficial in cases such as anesthesia and hemostasis. The hydrogels successfully demonstrated outstanding biological performance while carrying out the in vitro cytotoxicity on 3T3 cells, indicating the applicability of the hydrogel in the biomedical field.

4DP, up until this moment, is more of a concept rather than a large-scale go-to solution. The technology is somehow not still optimized for the upscaling, nor are the smart biomaterials available widely enough to facilitate the process; however, 4DP is one of the promising future research areas in TE, among other possible applications.

### 7.3. Organ-on-a-Chip (OOAC)

Perhaps the rapid and recent milestones achieved in the fields of organoids and microfluidic represent the main driving factor in pushing TE forward. An organoid is a miniaturized 3D multicellular structure fabricated in vitro from stem cells to mimic its in vivo analog, enabling us to study different physicochemical and physiological aspects of the target organ when exposed to certain treatment or stimulus in vitro [249,250]. Studying the stimulus effect and organ response vastly reduces the risk of failure post clinical trials.

On the other hand, microfluidic refers to the science that deals with the manipulation of fluids at the microscale level [251]. Advances in both organoids and microfluidic led to the development of organ-on-a-chip (OOAC) and body-on-a-chip (BOAC)/human-on-a-chip (HOAC) technologies. OOAC is an example of the integration of both fields for the creation of an exact replica of an in vivo organ while simulating the surrounding physiological environment [252]. It could be seen as a minute living tissue supported on a micro physiological device (chip). Complications in the ex vivo approach related to simulating in vivo conditions are reduced to a minimum with the development of OOAC. The improved mimicry has to do with the allowance of biofluids to pass into or through the organoid. BOAC or HOAC is adding other benefits to TE since many tissues from different organs are placed on a microfluidic chip. This allows studying disease etiology, the effect treatment and its metabolic byproduct have on many organs at once, and even anticipate possible adverse effects as many of those are evident at sites other than the target site [253,254,255,256]. For that and more, in World Economic Forum in 2016, OOAC was marked as one of the top 10 rising technologies and gained much interest for implementation in TE [251].

## 8. Challenges and Conclusions

Despite the major milestones accomplished in the field of TE and fabrication of scaffolds, there is still a major gap between the focus areas in literature and some key points if a broad application is desired. For example, most of the ongoing research focuses on the implantation of scaffolds of critical sizes with major neglection of large size tissue repair. The reason for the emergence of TE in the first place was to overcome the shortcomings of organ transplantations, and the focus on only certain size ranges is a major overlook until the moment.

Most scaffolds suffer from the problem of migration away from the implanted sites. To solve this, crosslinkers are usually added to the equation. However, their safety is a concern, and consequently, light must be shed on how to stabilize the scaffolds in their target site in the safest possible way.

In the case of non-replicating tissues, such as cardiac tissue and nerve cells, the direction is to go with the ex vivo approach of TE. That requires ex vivo cell propagation and tissue formation in an environment that perfectly matches its analog natural one. Despite the progress made in this field, reproducibility is troublesome since perfect mimicry of all the factors ex vivo is a very complicated task.

In scaffolds fabrication, large-scale production, as well as scaffold stability are still critical issues that require attention. There are still concerns about the safety of materials used in AM techniques, particularly in the long term. The cost-effectiveness of 4D scaffolds is still questionable.

It is true that we have come through a very long road in TE and scaffold manipulation, but the journey is far from over. As long as science is evolving around us, scientists will always provide the necessary means to overcome the shortcomings or further optimize the output of TE. The ultimate goal is to picture TE as a mainstream, feasible approach to organ failure with high success rates rather than the ongoing risky organ transplantation, and for this, further research is definitely needed.

## Figures and Tables

**Table 1:** Examples of biomaterials used in tissue engineering highlighting key advantages and disadvantages.

Biomaterial	Category	Advantages	Disadvantages	References
**Mg**	Metal, Biodegradable	High tensile strength Lightweight implant Comparable elastic modulus to that of bones	Excessive corrosion in biological fluid Release Mg ions on corrosion causing premature implant failure	[45,100,101]
**Ta**	Metal, Non-biodegradable	Exceptional corrosion resistance Biocompatible Enhance osseointegration	High elastic modulus High melting point, difficult to process	[45,102,103]
**Collagen**	Natural Polymer, Polypeptides	Rough surface Low immunogenicity Low toxicity	Susceptible to contraction and deformation Unstable in aqueous surroundings	[104,105]
**Gelatin**	Natural Polymer, Polypeptides	Lack of antigenicity Easily accessible functional groups for surface modification Its byproducts are nontoxic	Poor mechanical stability and low elasticity under physiological conditions	[106,107]
**Chitosan**	Natural Polymer, Polysaccharides	Anti-inflammatory, Antibacterial activities Nontoxic Enhances wound healing and tissue regeneration	Low mechanical resistance Unstable with uncontrollable dissolution	[2,57,105]
**Hyaluronic** **acid**	Natural Polymer, Polysaccharides	Promotes wound healing and fibroblast proliferation Bacteriostatic activity Non-immunogenic, nontoxic	Rapid in vivo degradation High viscosity	[108,109]
**PLA**	Synthetic Polymer, Polyester	Easily biodegradable with nontoxic byproducts Suitable mechanical properties Biocompatible	Hydrophobic with poor cell attachment Lack of thermal stability, degrades above 200 °C	[110,111]
**PLGA**	Synthetic Polymer, Polyester	Controllable biodegradability Biodegradable with faster degradation rate than PLA and PGA	Poor osteoconductivity Suboptimal mechanical strength	[112,113]
**PEG**	Synthetic Polymer, Polyol	Low immunogenicity and antigenicity Easily modifiable Biocompatible, rapidly cleared	Bioinert Non-biodegradable ∙	[114,115]
**HAp**	Ceramic, Biodegradable	Excellent resemblance to the natural HAp Osteoconductive activity Biocompatible and bioresorbable Suitable carrier for growth factors and osteoblasts	Brittle Poor mechanical strength	[116,117]
**Zirconia**	Ceramic, Bioinert	High fracture toughness Biocompatible Osteoconductive	Undergoes spontaneous transformation to the monoclinic phase causing surface instability and microcracking	[118,119]

**Table 2:** Examples of drug-loaded scaffolds showing their composition and fabrication technique.

Drug	Applications	Composition	Fabrication Technique	Key Findings	Morphological Features of The Scaffold	References
Vancomycin hydrochloride and gentamicin sulfate (Antibiotics)	Treatment of osteomyelitis generated during the implantation of the scaffolds in the defected bone.	Collagen and magnesium-doped hydroxyapatite.	Scaffolds were fabricated via lyophilization technique followed by dehydrothermal crosslinking method (chemical crosslinking).	Scaffolds possessed high porosity (>90%) with interconnected macro- and micropores Increasing the amount of magnesium-doped hydroxyapatite created more binding sites with the loaded drugs and hence more sustained drug release (up to 20 days) Antimicrobial activities of the drugs were preserved after scaffolds loading	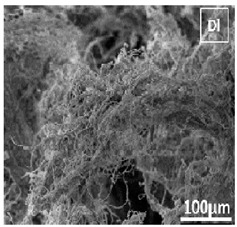 ESEM image of the scaffold	[224]
Ketoprofen (Non-steroidal anti-inflammatory drug)	Bone fractures and diseases	Poly(ε-caprolactone) and ammonium bicarbonate (porogen)	Scaffolds were prepared via supercritical foaming technology using a solid porogen that was removed without the need for solvent leaching	The scaffolds were porous with interconnected pores, where the % porosity was around 63% Increasing porogen amount enhanced the scaffold macroporosity as well as decreased its mechanical strength Scaffolds possessed excellent cytocompatibility after 2 days confirming the complete removal of the porogen	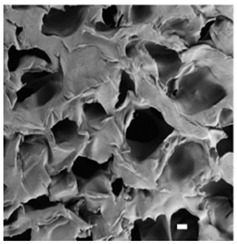 SEM image of the scaffold	[225]
Rifampicin (Antibiotic)	Bone tissues restoration	Biphasic calcium phosphate as scaffold matrix and poly(ε-caprolactone) or poly (ester urea) as coating materials	Hydrothermal treatment of cuttlefish bone into biphasic calcium phosphate. Coating of the scaffolds was carried out by simple dipping in the polymer organic solution under vacuum	The type of the scaffold polymer coating material significantly affected the drug release. Poly(ε-caprolactone)-coated scaffolds showed higher burst release compared to poly (ester urea)-coated ones. The same effect was detected after 6 days Poly(ε-caprolactone)-coated scaffolds succeeded in eradicating *E. coli* and *S. aureus* after incubation for 72 h The polymer-coated scaffolds augmented cellular adhesion and proliferation against hMSCs cells with minimal cytotoxicity	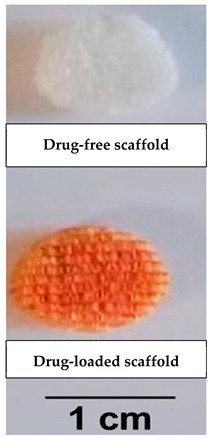 External morphological features of drug-free and drug-loaded scaffolds	[226]
Dexketoprofen trometamol (Non-steroidal anti-inflammatory drug)	Chronic wounds	Chitosan, polyvinyl alcohol, gelatin, citric (pH modifier) and benzoic (antimicrobial activity) acids	Solvent casting technique followed by oven-drying	Scaffolds lacking the addition of polyvinyl alcohol or gelatin showed surface pores in SEM images Chitosan scaffolds crosslinked using gelatin exhibited the most sustained drug release profile Future research to study the effect of the selected scaffolds on cellular proliferation, as well as the investigation of the anti-inflammatory effect, would be carried out	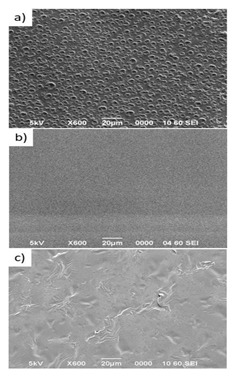 SEM images of chitosan scaffolds (a) lacking the addition of crosslinkers, (b) crosslinked with polyvinyl alcohol, and (c) crosslinked with gelatin	[227]
Insulin-like growth factor-1 (Chemotactic migration of osteoblasts)	Promotion of bone repair and regeneration	Sodium alginate, poloxamer 407 and silk fibroin were used for the preparation of the hydrogel. Mesoporous bioactive glass nanoparticles were used for loading insulin-like growth factor-1	Thermosensitive hydrogels were prepared using alginate-poloxamer copolymer. Silk fibroin was added to enhance the mechanical strength of the formed hydrogels and was transformed into hydrogel using H_2_O_2_ (cross-linker)	Concentrations of 12% *w*/*w* or more of alginate-poloxamer copolymer succeeded in having clear sol-gel transition Mesoporous bioactive glass nanoparticles were characterized with high pore volume of 0.49 ± 0.02 or 0.61 ± 0.03 mL/g (according to the preparation method), which resulted in enhanced loading efficiency of insulin-like growth factor-1 up to 60% The growth factor release from the prepared hydrogels followed more sustained release behavior when it was loaded in the bioactive glass compared to that directly dispersed in the hydrogel	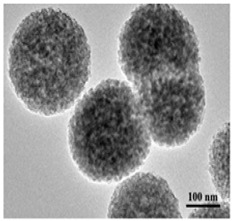 TEM image of mesoporous bioactive glass nanoparticles 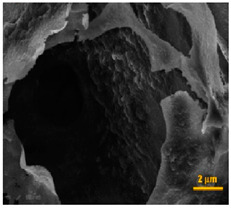 SEM image of dry hydrogel showing porous structure as well as the mesoporous bioactive glass nanoparticles or their aggregates attached to the porous walls	[228]
Copper ions (Antibacterial and remineralizing)	Dental composites to mitigate secondary caries	Resin composites made up from bisphenol A–glycidyl methacrylateand triethylene glycol dimethacrylate and loaded with copper-doped mesoporous bioactive glass nanospheres along with silica fillers	Copper-doped mesoporous bioactive glass nanospheres were fabricated using	The reinforcement of copper-doped mesoporous bioactive glass nanospheres might be ascribed to the combinatory effect of both the resin and the silica fillers, which delayed the deterioration of the bioactive glass in water up to 28 days The developed composite is a propitious path for the fabrication of antibacterial and ion-releasing matrix	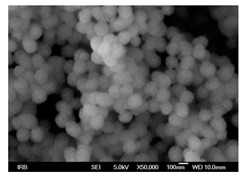 SEM of copper-doped mesoporous bioactive glass showing spherical particles with average diameter of 100 nm	[229]

## Data Availability

Data sharing is not applicable to this article as no new data were created or analyzed in this study.
